# Clinical-Pathological Conference Series from the Medical University of Graz: Case No 159: 52-year-old patient with psoriasis and arthralgia of the finger joints

**DOI:** 10.1007/s00508-016-1010-0

**Published:** 2016-06-30

**Authors:** Elisabeth Fabian, Dietmar Schiller, Winfried Graninger, Cord Langner, Johannes Frei, Helmut Schoellnast, Vedat Alibegovic, Rudolf Stauber, Rainer Schoefl, Guenter J. Krejs

**Affiliations:** 1 Division of Gastroenterology and Hepatology, Department of Internal Medicine III, Medical University of Vienna, Vienna, Austria; 2 Department of Internal Medicine IV, Elisabethinen Hospital, Linz, Austria; 3 Division of Rheumatology and Immunology, Department of Internal Medicine, Medical University of Graz, Graz, Austria; 4 Department of Pathology, Medical University of Graz, Graz, Austria; 5 Department of Radiology, Elisabethinen Hospital Linz, Linz, Austria; 6 Division of General Diagnostic Radiology, Department of Radiology, Medical University of Graz, Graz, Austria; 7 Department of Pathology, Elisabethinen Hospital, Linz, Austria; 8 Division of Gastroenterology and Hepatology, Department of Internal Medicine, Medical University of Graz, Auenbruggerplatz 15, 8036 Graz, Austria

**Keywords:** Hemochromatosis, Arthralgia, Cystic bone changes

## Presentation of case

### *Dr. D. Schiller*:

Since age 18 the patient had suffered from mild psoriasis that was treated with intermittent courses of topical corticosteroids. For the past 5 years he had complained of pain in his finger joints aggravated by manual work. The patient worked in an office and denied morning stiffness, back pain, repetitive strain injury related to leisure activities, enthesiopathy, gout or inflammation of the eyes. His family history was unrevealing. For the past 3 years he had taken lisinopril and hydrochlorthiazide for arterial hypertension.

An ileocolonoscopy due to diarrhea 5 months prior to admission showed diffuse mucosal erythema of the descending and sigmoid colon. Biopsies showed nonspecific inflammation with slightly increased eosinophils in the lamina propria. Esophagogastroduodenoscopy with gastric and duodenal biopsies yielded normal results; the hydrogen breath test suggested lactose intolerance. His diarrhea resolved with a lactose-poor diet and 5‑aminosalicylic acid (5-ASA, 3 g per day for 3 months).

After an exacerbation of his psoriasis had persisted for 4 weeks he was admitted with pustules and progressive diffuse erythrodermia covering about 80 % of his body. The pustules were round with desquamating margins. On physical examination he was afebrile; his weight was 83 kg and height 178 cm. The finger- and toenails appeared normal. Several finger joints and the metacarpophalangeal (MCP) joints of both index and middle fingers were swollen and indurated without hyperthermia or erythema.

Routine laboratory tests, including a complete blood count, liver and renal function tests, erythrocyte sedimentation rate, C‑reactive protein (CRP) as well as antinuclear antibodies (ANA), anti-cyclic citrullinated peptide (anti-CCP) antibodies, immunoglobulin (Ig) G, IgA, IgM and anti-tissue-transglutaminase antibodies were normal or negative. Chest radiography and abdominal ultrasound were unremarkable. Plain radiographs of the hands showed irregular margins of the 2nd and 3rd MCP joints bilaterally with some small subchondral cysts and osteophytes on the radial side. Cystic changes were also seen in the carpal bones bilaterally, most pronounced in the os triquetrum and lunatum.

A diagnostic procedure was performed.

### *Dr. C. Langner*:

Histological examination of the colon biopsies mentioned in the protocol revealed active inflammation of the lamina propria with neutrophil cryptitis and mild eosinophilia. Since these histopathological features are not very specific and can be found within a wide spectrum of diseases, histology does not provide a clear clue for an underlying disease.

### *Dr. H. Schoellnast*:

**Fig. 1:**
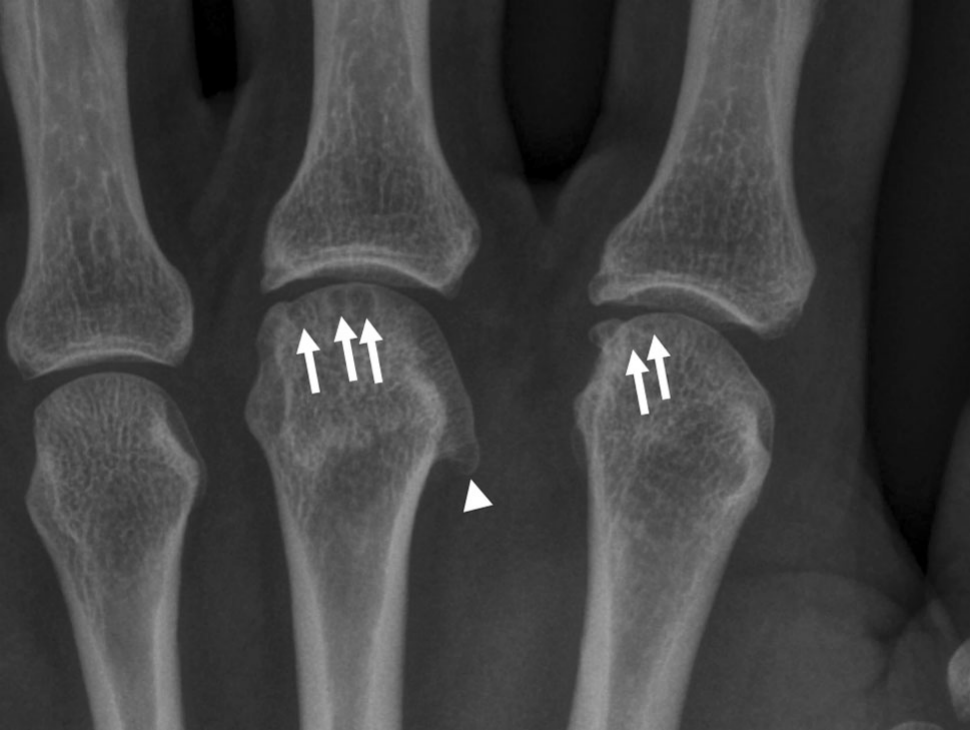
Radiography of the left hand with subchondral cysts and osteophytes in the 2nd and 3rd MCP joints (*arrows*), and overhanging and hook-like radial osteophyte in the 3rd metacarpal head (*arrow head*). The margins of these joints are irregular but the joint spaces were unremarkable. These findings were the same for both hands

Radiography of the hands showed a remarkably symmetric pattern with subchondral cysts and osteophytes in the 2nd and 3rd MCP joints of both hands. There were hook-shaped radial osteophytes, most pronounced in the metacarpal head. Bilaterally, the margins of these joints were irregular; the joint spaces were unremarkable. Except for some further small subchondral cystic lesions in single carpal bones that were most pronounced in the triquetum and lunatum, radiography was unremarkable (Fig. 1).

## Differential diagnosis

### *Dr. W. Graninger*:

The patient had a medical history of psoriasis with mild course so far and arthralgia of the finger joints, but required hospitalization when he presented with this acute exacerbation of his psoriasis featuring round pustular rash, desquamation, and progressive diffuse erythrodermia affecting about 80 % of his skin, and joint pain in the hand (several finger joints and the 2nd and 3rd MCP joints of both hands were swollen and indurated but without erythema and hyperthermia). At first glance, these findings suggest psoriatic arthritis, which is a symmetric, progressive, chronic inflammatory joint disorder with heterogeneous clinical features that may include plaque psoriasis, joint inflammation, enthesitis (inflammation at the sites where tendons or ligaments insert into the bone), dactylitis (diffuse swelling of the fingers and toes), and abnormal bone turnover. Psoriatic arthritis affects up to 0.5 % of the population with equal prevalence in males and females [1]. Methotrexate and infliximab are standard medications for psoriatic arthritis. In this patient, psoriatic arthritis is, however, questionable because (1) there are no histological findings confirming the diagnosis of plaque psoriasis and diffuse pustulous erythrodermia, (2) there is no evidence from laboratory data (normal erythrocyte sedimentation rate and normal CRP levels are atypical for psoriatic arthritis when psoriasis affects 80 % of the skin; however, cases of erythrodermia due to Sézary syndrome and seronegative polyarthritis with normal laboratory results have been described [2]), and (3) the patient’s finger- and toenails were unremarkable but would be expected to show typical changes with psoriatic arthritis. For the right diagnosis we, therefore, have to look at additional facts. Five months prior to admission due to exacerbation of psoriasis and arthralgia of the finger joints, the patient had suffered from diarrhea and was diagnosed with lactose intolerance and acute colitis with eosinophils in the lamina propria. Eosinophilic colitis is a nonspecific diagnosis that can be found in a wide spectrum of diseases. In this case, medical treatment with 3 g 5‑ASA per day for 3 months successfully resolved the gastrointestinal symptoms. Unfortunately, a follow-up colonic biopsy is not available, so it is not clear whether the mucosa recovered under this treatment. Adverse drug reactions with 5‑ASA [3] are rare, and it is unlikely that our patient’s dermatological symptoms represented an adverse drug reaction because the acute exacerbation of psoriasis and erythrodermia did not develop until 2 months after 5‑ASA was discontinued.

Focusing on the arthralgia of finger joints, it has to be emphasized that the 2nd and 3rd MCP joints on both hands were mainly affected. This is a very specific finding because osteoarthritis of the hand usually affects distal and proximal, interphalangeal and basal joints of the first ray, but not MCP joints. Further, the MCP joints of both index and middle fingers were described as swollen and indurated but without erythema and hyperthermia, which might be due to synovitis. Sonography of the hands would be helpful here and would allow differentiation between degenerative changes of the joints, psoriatic arthritis, perisynovitis, and other rheumatological diseases [4]. In addition to subchondral cysts and osteophytes, radiography also revealed irregular margins of the 2nd and 3rd MCP joints of both hands. These manifestations in the MCP joints are specifically found in Dietrich’s disease, an avascular necrosis. Causes for avascular necrosis include trauma (fracture or dislocation), caisson disease, hemoglobinopathies such as sickle cell disease, pregnancy, radiotherapy, connective tissue disorders, renal transplantation, corticosteroid excess (both endogenous and exogenous), pancreatitis, gout, Gaucher’s disease, and alcohol abuse [5]. However, since none of these causes seem to apply to our patient, I would exclude Dietrich’s disease as a differential diagnosis. Subchondral cysts as found here are typical for degenerative joint disease, rheumatoid arthritis, calcium pyrophosphate dihydrate (CPPD) crystal deposition disease, avascular necrosis, hemophilia, sickle cell disease, multiple myeloma, amyloidosis, hemochromatosis, Wilson’s disease, and hyperparathyroidism [6]. Since routine laboratory tests, including complete blood count, liver and renal function tests, erythrocyte sedimentation rate, CRP, ANA, anti-CCP antibodies, IgG, IgA, IgM, and anti-tissue-transglutaminase antibodies were normal or negative, most of those diseases can be excluded. However, the radiologic constellation with characteristic changes, subchondral cysts and osteophytes primarily affecting the 2nd and 3rd MCP joints of both hands is a typical finding in hemochromatosis. This is an iron metabolism disorder characterized by increased intestinal iron absorption and progressive deposition in organs and tissues resulting in injury and functional impairment, particularly in the liver, pancreas, heart, joints, and pituitary [7]. Laboratory findings include increased ferritin levels and enhanced transferrin saturation. Schumacher first recognized the relationship between hemochromatosis and arthritis in 1964 [8]. Joint pain is present in about 30 % of affected patients and is sometimes reported as the first symptom. Typically, the 2nd and 3rd MCP joints are involved but virtually any joint can be affected with signs and symptoms of osteoarthritis [9, 10]. Moreover, hook osteophytes along the radial aspect of the distal metacarpals are frequently found in arthropathy due to hemochromatosis. These hook osteophytes can also be seen in CPPD crystal deposition disease but are more prevalent in hemochromatosis. Generally, crystals are not associated with hemochromatosis arthropathy, but some patients may present with apatite and CPPD crystals, in which case arthritis mimics pseudogout (Table 1) [11, 12]. To further differentiate between hemochromatosis and CPPD crystal deposition disease, the following has to be considered: Both diseases involve the radiocarpal and midcarpal compartments of the wrist with diffuse joint space narrowing and intraarticular and periarticular calcifications, which are not seen with osteoarthritis. What helps to differentiate between hemochromatosis and CPPD crystal deposition disease is the joint space. In hemochromatosis arthropathy there is uniform loss of joint space at all the MCP joints including those of the ring and little fingers, while in CPPD crystal deposition disease there is narrowing predominantly of the index and the middle finger MCP joints [13]. Hook osteophytes may also be present on radiographs in cases of erosive arthropathy, but in erosive arthropathy distal interphalangeal joints are affected and clinically, these hook osteophytes have a distinct brown, wart-like appearance [14]. Since the pathological changes in this patient’s finger joints and his erythrodermia are not features of erosive arthropathy, this diagnosis can be ruled out.

**Table 1:** Features of hemochromatosis arthropathy and other common joint conditions [11, 12]

	Hemochromatosis	Osteoarthritis	Rheumatoid arthritis	CPPD crystal deposition disease
Age of onset	<50 years	>50 years	>45 years^a^	Usually >60 years
Chondrocalcinosis	Common	Rare	Rare	Very common
MCP involvement	Very common	Rare	Very common	Common
Signs of synovitis	Occasional	Rare	Very common	Episodic
Marginal erosions	Very rare	Very rare	Common	Very rare

*MCP* metacarpophalangeal, *CPPD* calcium pyrophosphate dehydrate

^a^Incidence appears to increase in men after age 45; in women incidence increases to a plateau and then appears to decline after age 75–80

Due to the clinical presentation, sarcoidosis or osteitis cystica multiplex (Jüngling’s disease), a slowly progressive form of osteitis with cystic changes extending into surrounding soft tissue in the acral regions (fingers, toes, nose) might also be considered as diagnosis here, if it were not for our patient’s normal chest radiographs.

Taken together, we have three important findings in this case: cystic arthropathy in the 2nd and 3rd MCP joints, pustulous psoriasis affecting 80 % of the skin, and eosinophilic colitis. The radiological findings strongly suggest arthropathy associated with hemochromatosis. Further laboratory data such as ferritin, transferrin saturation, and search for hemochromatosis gene mutations are needed to confirm this diagnosis. Moreover, I would suggest performing a skin biopsy to provide more information on his skin disease with pustulous psoriasis and erythrodermia.

## Dr. W. Graninger’s diagnosis

Arthropathy due to hemochromatosis.

## Discussion of case

### *Dr. G.J. Krejs*:

Our patient did indeed suffer from hemochromatosis-associated arthropathy. The radiologist (J. F.) gave the first hint pointing toward the right diagnosis by noting the characteristic changes in the 2nd and 3rd MCP joints of both hands. Dr. Schoellnast will explain these remarkable radiological features of hemochromatosis arthropathy in detail.

### *Dr. H. Schoellnast*:

Radiological features of hemochromatosis arthropathy of the hands are usually seen bilaterally and include joint space narrowing, cyst formation, and osteophytes in the 2nd and 3rd MCP joints and less frequently in the 4th and 5th MCP joints as well. Typical findings in hemochromatosis are hook- or beak-shaped osteophytes along the radial aspect of the 2nd and 3rd metacarpal heads, features that are not seen with osteoarthritis. MCP joint manifestations are common in hemochromatosis although other joints such as the wrists, knees, hips, shoulders, and ankles may also be affected [9, 10]. In hemochromatosis arthropathy, joints are not usually affected by the degenerative or erosive changes that are typical for other polyarthropathies of the hand including psoriatic arthritis. The location of the pathological changes occurring with various arthropathies is characteristic for particular diseases. While hemochromatosis arthropathy typically affects the 2nd and 3rd MCP joints, other arthropathies typically affect distal interphalangeal and proximal interphalangeal joints and the basal joint of the first ray, but not the MCP joints. In this patient, the margins of the affected MCP joints were slightly irregular in both hands but there were no signs of degenerative or erosive disease. The joint spaces were unremarkable, though they can also show characteristic changes, i. e. uniform loss of joint space in all the MCP joints (2nd–5th). This feature may also help to differentiate between hemochromatosis arthropathy and other pathological changes such as CPPD crystal deposition disease, where MCP joint space narrowing is less prevalent and predominantly affects the 2nd and 3rd MCP joints. In hemochromatosis arthropathy the 4th and 5th MCP joints are also more often narrowed [13, 15]. Since their features substantially overlap (including chondrocalcinosis, radiographic appearance, and to a large degree distribution of affected joints), it may be very difficult to differentiate between hemochromatosis arthropathy and CPPD crystal deposition disease. Radiologically, CPPD crystal deposition disease in the wrist is more likely to cause scapholunate dissociation, which is a loss of the normal anatomical articulation of the scaphoid with the lunate, manifesting as a malalignment of these bones on an anteroposterior projection [13, 15].

### *Dr. D. Schiller*:

In this patient the radiologist suspected the diagnosis of hemochromatosis arthropathy, which could be confirmed by the following parameters: serum iron 307 µg/dl (normal: 37–170 µg/dl), transferrin saturation 100 % (normal: 20–60 %), serum ferritin 2080 ng/ml (normal: 11–264 ng/ml). HFE gene analysis showed a homozygous C282Y mutation in the HFE gene. Hemochromatosis is a genetic disorder of iron metabolism leading to increased enteric absorption of iron and progressive total body iron overload. Excessive iron stores and deposition of hemosiderin can cause tissue damage and dysfunction in a number of organ systems [16]. The diagnosis of hemochromatosis is sometimes delayed because the symptoms are nonspecific and overlap with those of other conditions. Joint manifestations are common and as in our patient may lead to the diagnosis of hemochromatosis. However, as discussed by Dr. Graninger, it has to be considered that joint findings in hemochromatosis may mimic those seen in other common rheumatologic conditions such as osteoarthritis and rheumatoid arthritis. Chondrochalcinosis or CPPD crystal deposition disease (pseudogout) is frequently found in patients with hemochromatosis and may be the first manifestation. Routine genetic screening of these patients for hemochromatosis has been shown to be cost effective because it allows early identification and treatment [17]. The clinical features of hemochromatosis result from damage to various organs due to iron overload and appear earlier in affected men than in women [18, 19]. Since diagnostic tools have improved, the classic presentation with cirrhosis, diabetes mellitus, and bronze skin is rarely seen today; the most common initial symptoms are fatigue, malaise, and joint pain [18]. Hepatic iron overload leads progressively to fibrosis and cirrhosis with ultimately an increased risk of hepatocellular carcinoma. Deposition of iron in islet cells of the pancreas may result in diabetes mellitus, while cardiac iron deposition may cause cardiomyopathy with arrhythmia and congestive heart failure. Iron overload in the pituitary gland can lead to hypogonadotropic hypogonadism, hypothyroidism, or panhypopituitarism. Hypopituitarism is characteristic for juvenile hemochromatosis (hemochromatosis type 2) and is often its first manifestation [20]. Moreover, patients with iron overload, including those with hereditary hemochromatosis, are at risk for a number of bacterial infections (e. g., *Yersinia enterocolitica, Listeria monocytogenes, Vibrio vulnificus*) [21–23].

After our patient was diagnosed with hemochromatosis, therapeutic phlebotomy was initiated. Phlebotomy is the mainstay of treatment, both for initial depletion of excess iron stores and for subsequent maintenance of normal total body iron. There are no standardized guidelines but the efficacy of phlebotomy has been reported with an induction regime of removal of 500 ml of whole blood weekly (i. e. 200–250 mg iron) until the hematocrit falls 2–4 % below baseline and ferritin levels normalize to within 25–50 µg/l. Lifelong maintenance phlebotomy involves removal of 500 ml of whole blood every 2–4 months in men or every 3–12 months in women before menopause. This therapy usually allows maintenance of the serum ferritin level within the lower normal range. If iron parameters increase, suggesting inadequate iron removal, the frequency of phlebotomy has to be adapted. In the absence of contraindications, phlebotomized blood can be used for blood donation [20].

### *Dr. G.J. Krejs*:

Progressive hemochromatosis is potentially fatal; before the introduction of phlebotomy therapy in the 1950s, mean survival after diagnosis was only 1.5 years. Today, decompensated liver cirrhosis and hepatocellular carcinoma represent the main causes of hemochromatosis-related mortality, mostly due to delayed diagnosis [24, 25]. Phlebotomy is a highly effective therapeutic approach in mild to moderate hemochromatosis and adequately treated patients have a normal life expectancy [26]. Especially when the disease is mild, some clinical features may be reversed with adequate therapy. However, unlike the other organopathies observed with hemochromatosis, arthritis does not regress with treatment [27, 28] and it is suggested that severity of hemochromatosis arthropathy correlates with iron stores [29]. Iron is known to catalyze oxidative reactions with subsequent formation of reactive oxygen species that can cause inflammation and tissue damage. Moreover, iron stimulates DNA synthesis in synovial cells with an additive effect on cell proliferation, together with cytokines such as IL-1ß and TNF-α [30]. This suggests that iron has a pivotal role in modulating the activity of arthropathies and may also be a relevant factor in rheumatological diseases [31].

As mentioned, hemochromatosis is also associated with an enhanced risk for various infectious diseases because iron is an essential growth factor for many bacteria and increases bacterial virulence [32, 33]. Moreover, data indicate that iron has a significant unfavorable influence on the human gut microbiota and so may indirectly trigger intestinal inflammation in patients with hemochromatosis [32, 34]. This might have been a relevant contributing factor to our patient’s nonspecific colonic inflammation. We think that he had some form of infectious colitis that cleared. Recently, clinical observations highlight the relationship between iron and *Helicobacter pylori *infection. This infection is frequently associated with refractory iron deficiency anemia that responds favorably to *Helicobacter pylori* eradication [35, 36]. *Helicobacter pylori *can contribute to low iron status by different mechanisms. These include bacterial uptake of dietary and host-derived (lactoferrin, saturated transferrin, hemoglobin) iron, which increases virulence and may decrease human iron status. Increased virulence may accelerate development of atrophic gastritis, resulting in hypo- or achlorhydria and reduced secretion of vitamin C from the gastric epithelium, which also impairs iron uptake. Further, low iron status contributes to induction of gastric hepcidin, thus, promoting development of peptic ulcer [37, 38].

The influence of gastric acid secretion on iron absorption is also emphasized by the increased notion that achlorhydria in atrophic gastritis may be the sole cause of iron deficiency anemia [39, 40]. Therapeutic hypochlorhydria caused by proton pump inhibitors may thus reduce the frequency of phlebotomy in patients with hemochromatosis [41].

### *Dr. D. Schiller*:

The potential gain of information from a liver biopsy was discussed with the patient and he agreed to a blind liver biopsy, which was carried out without complications.

### *Dr. C. Langner*:

Liver biopsy is very informative in selected patients with hemochromatosis but is not standard since it usually will not provide further information (cirrhosis is not likely to be present in patients with a serum ferritin level lower than 1000 ng/ml) or prompt a change in therapy [42]. Since ferritin exceeded 1000 ng/ml in this case, biopsy was recommended to evaluate whether and/or to what extent the liver had already been affected by iron overload.

**Fig. 2:**
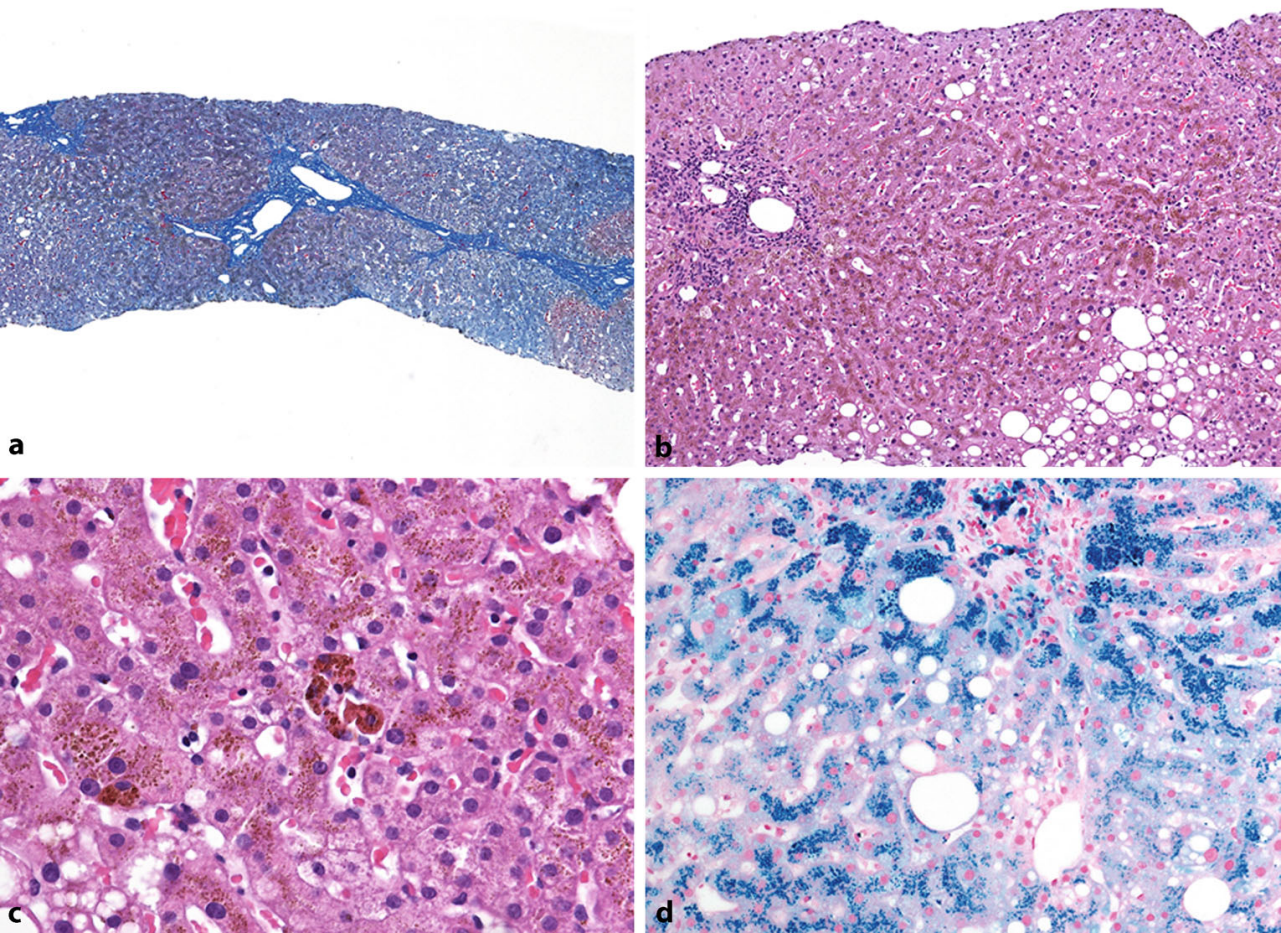
Liver histology shows preserved lobular architecture with mild portal fibrosis and occasional porto-portal bridging, but no cirrhosis (**a**, *trichrome*). There is mild zone 3 steatosis and mild lobular necroinflammatory activity (**b**, *H&E*). On higher magnification, brownish granular pigment is seen within hepatocytes and occasional Kupffer cell nodules (**c**, *H&E*). Prussian blue staining identifies the pigment as accumulation of iron, i. e. hemosiderin (**d**)

The liver biopsy showed mild enlargement of the portal fields with occasional porto-portal bridging, but no cirrhosis. There was mild necroinflammatory activity within the lobular parenchyma as well as mild steatosis. Iron stains showed marked accumulation of iron in hepatocytes and occasional macrophages within the portal fields (Fig. 2).

### *Dr. R. Stauber*:

Generally, besides liver biopsy, Fibroscan™ could also be applied to grade fibrosis due to hepatic iron overload in hemochromatosis. Since this method is not well standardized for this disease it can only provide limited information on the effects, if any, of iron overload. Thus, in some patients with hemochromatosis, liver biopsy is necessary to obtain further diagnostic and prognostic information.

Hemochromatosis is an inherited disorder of iron metabolism leaving the patients unable to effectively regulate their iron absorption and so resulting in progressive accumulation of iron. Several different types of the disease have so far been described. In these individuals absorption of both inorganic and heme iron is increased [43] because basolateral efflux of iron from the enterocytes is inappropriately regulated [44, 45]; most importantly hepcidin levels are very low in patients with hemochromatosis type 1 (mutation of the HFE gene, MIM #235200) [46]. In a normal individual an increase in body iron levels would lead to increased hepcidin expression and this in turn would decrease iron absorption by reducing basolateral efflux. Such an increase in hepcidin does not occur in patients with HFE-associated hemochromatosis, so that their iron absorption continues in the face of body iron overload. Mutations of HFE, hemojuvenil, hepcidin, transferrin receptor 2 (TfR2), Ireg1, transferrin, ceruloplasmin and ferritin all lead to some form of iron overload [47]. Of these, mutations in the HFE gene located on chromosome 6 are by far the most common [48] and >80 % of patients with hemochromatosis are homozygous for HFE mutation C282Y (rs1800562, G845A) [49, 50]. HFE-associated hemochromatosis, the most common type in adults, is regarded as the most prevalent autosomally inherited genetic disease of Caucasians with an allele frequency of C282Y of 6.2 % and a homozygosity frequency of 0.38 % in a pool of 127,613 individuals. However, there is considerable variation across Europe with allele frequencies of >10 % in Ireland and 0–3 % in Mediterranean regions [27]. In the US, homozygosity for the C282Y mutation accounts for most cases of hemochromatosis followed by H63D and S65C mutations. Heterozygosity of C282Y and H63D is seen only infrequently. Penetrance of C282Y is, however, low. A population-based study revealed mildly elevated liver enzymes or liver disease in <10 % of C282Y homozygotes and the estimated prevalence of clinical hemochromatosis was as low as 1 % [51]. Other studies reported liver disease in up to 16 % of C282Y homozygotes [52]. In addition to the particular genetic variant, penetrance of the mutation and environmental factors such as diet, alcohol consumption, coexisting chronic viral hepatitis, and host factors such as gender, age, and obesity may influence the course of hemochromatosis-associated fibrosis and cirrhosis [27, 53]. Patients with HFE-associated hemochromatosis absorb excessive amounts of iron from birth, which entails excessive iron accumulation in parenchymal cells of the liver, heart, and endocrine organs [53]. While a normal adult male may have 3.5 g of storage iron in his body, patients with hemochromatosis may reach 20–30 g or more before clinical symptoms occur [18]. In my experience patients typically first present around age 50 due to hepatic symptoms; about 1/3 of affected patients have joint manifestations and are first seen by rheumatologists. Sometimes chondrocalcinosis and subsequent profound joint destruction of the knees, hips, or shoulders may have already necessitated joint replacement before the diagnosis of hemochromatosis. Hemochromatosis type 2, also defined as juvenile hemochromatosis, is divided into type 2A (mutation of the hemojuvenile gene, HJV, MIM #602390) and type 2B (mutation of the hepcidin antimicrobial peptide gene, HAMP, MIM #613313). Mutations in the transferrin receptor 2 (TFR2) gene were identified as a cause of hemochromatosis type 3 (MIM #604250) [54]. Hemochromatosis types 4 and 5 are associated with mutations of the ferroportin (Ireg 1) gene (SLC40A1, MIM #606069) and mutations of the H ferritin gene (FTH1, MIM #615517), respectively [55]. Genes associated with iron metabolism represent obvious candidates for mutations that lead to iron overload and disease phenotype in hemochromatosis. Further investigations will determine the role of each of these genes and their mutations and also of duodenal cytochrome b (DCYTB, CYBYRD1) and others in iron accumulation [47].

### Dr. G.J. Krejs:

This case clearly shows that diagnosis of hemochromatosis can sometimes be difficult and is often delayed for decades because of silent iron deposition without symptoms. Since the clinical features are nonspecific and the symptoms of hemochromatosis arthropathy overlap with those of other rheumatologic conditions, diagnosis is difficult even in symptomatic patients. Currently, our patient has regular therapeutic phlebotomy and he will surely also be screened regularly for hepatocellular carcinoma.

### Dr. W. Graninger:

In this case a history of psoriasis made it difficult to relate the joint symptoms to their true cause, hemochromatosis. Joint symptoms may be the first manifestation of hemochromatosis, especially when it is HFE-associated. Although it is sometimes difficult, there are both clinical and radiographic clues to differentiate between hemochromatosis arthropathy and other rheumatologic diseases. Rheumatologists should be aware of the characteristic clinical features and symptoms, and the typical radiologic findings in hemochromatosis arthropathy to correctly identify hemochromatosis patients as early as possible, thus, ensuring adequate treatment.

## Final diagnosis

Hemochromatosis arthropathy.
